# Pharmacological c-Jun NH_2_-Terminal Kinase (JNK) Pathway Inhibition Reduces Severity of Spinal Muscular Atrophy Disease in Mice

**DOI:** 10.3389/fnmol.2018.00308

**Published:** 2018-09-04

**Authors:** Roberta Schellino, Marina Boido, Tiziana Borsello, Alessandro Vercelli

**Affiliations:** ^1^Department of Neuroscience Rita Levi Montalcini, Neuroscience Institute Cavalieri Ottolenghi (NICO), University of Turin, Turin, Italy; ^2^National Institute of Neuroscience (INN), Turin, Italy; ^3^Department of Pharmacological and Biomolecular Sciences, University of Milan, Milan, Italy; ^4^Department of Neuroscience, IRCCS-Mario Negri Institute for Pharmacological Research, Milan, Italy

**Keywords:** motor neuron disease, MAPK, motor endplates, therapy, innervation, muscle, motor performance, apoptosis

## Abstract

Spinal muscular atrophy (SMA) is a severe neurodegenerative disorder that occurs in early childhood. The disease is caused by the deletion/mutation of the survival motor neuron 1 (SMN1) gene resulting in progressive skeletal muscle atrophy and paralysis, due to the degeneration of spinal motor neurons (MNs). Currently, the cellular and molecular mechanisms underlying MN death are only partly known, although recently it has been shown that the c-Jun NH_2_-terminal kinase (JNK)-signaling pathway might be involved in the SMA pathogenesis. After confirming the activation of JNK in our SMA mouse model (SMN2+/+; SMNΔ7+/+; Smn−/−), we tested a specific JNK-inhibitor peptide (D-JNKI1) on these mice, by chronic administration from postnatal day 1 to 10, and histologically analyzed the spinal cord and quadriceps muscle at age P12. We observed that D-JNKI1 administration delayed MN death and decreased inflammation in spinal cord. Moreover, the inhibition of JNK pathway improved the trophism of SMA muscular fibers and the size of the neuromuscular junctions (NMJs), leading to an ameliorated innervation of the muscles that resulted in improved motor performances and hind-limb muscular tone. Finally, D-JNKI1 treatment slightly, but significantly increased lifespan in SMA mice. Thus, our results identify JNK as a promising target to reduce MN cell death and progressive skeletal muscle atrophy, providing insight into the role of JNK-pathway for developing alternative pharmacological strategies for the treatment of SMA.

## Introduction

Spinal Muscular Atrophy (SMA) is a recessive autosomal neuromuscular disease that represents the most common fatal pathology in infancy. The disease is caused by the mutation or deletion of the survival motor neuron 1 (SMN1) gene, that leads to a progressive degeneration of spinal motor neurons (MNs), resulting in the atrophy of the muscles of the limbs and trunk, and lastly in death for respiratory complications (Lorson et al., 1999). Due to its defective splicing pattern, the highly homologous copy gene SMN2 generates an insufficient amount of functional SMN protein: therefore, the SMN2 copy number inversely correlates with the severity of SMA phenotype (Lorson et al., 2010), accounting for the presence of four main clinical SMA types (SMA I-IV), characterized by different age of onset and disease progression (Lefebvre et al., 1995).

Despite the genetic causes of SMA are well known, the mechanisms underlying MN death are still poorly understood. The low levels of SMN might probably lead to a selective activation of intracellular stress signaling pathways in MNs, initiating neurodegeneration (Burghes and Beattie, 2009; Genabai et al., 2015).

Thus, investigating the molecular and cellular mechanisms of cell death leading to MN degeneration could be extremely relevant to prevent and/or delay the disease progression. Indeed, although in the last years different translational approaches, both SMN-dependent (e.g., RNA-based modulation of SMN2) and SMN-independent (e.g., neurotrophic factors, stem cells), have been adopted to develop therapeutics for SMA patients (Lorson et al., 2010; Boido and Vercelli, 2016), the molecular pathways involved in neurodegeneration remain unknown.

Recently, Genabai et al. (2015) reported that the c-Jun NH_2_-terminal kinase (JNK) cascades ASK1/MKK4/7/JNK and MEKK1/MKK4/7/JNK are activated in spinal cord of SMA mice (SMNΔ7) and patients (Genabai et al., 2015): in particular, an increase in phosphorylation of the three JNK isoforms (JNK1, 2, 3) was visible in SMA phenotype and the brain-specific isoform JNK isoform 3 (JNK3) mediated the neurodegeneration caused by the low levels of SMN. By creating a double JNK3-SMNΔ7 knockout (KO) mouse model, the authors obtained a milder SMA phenotype, demonstrating that JNK pathway might represent a potential SMN-independent therapeutic target for the treatment of SMA.

Based on these results, here we investigated the possibility to impact on SMA progression by JNK inhibition: to this aim, we administered to SMNΔ7 mice (mouse model of intermediate SMA) a cell-penetrating JNK inhibitor (D-JNKI1) peptide, which selectively blocks the access of JNK to c-Jun and the other JNK-binding domain (JBD) dependent targets. The structure of the peptide and its competitive mechanism of inhibition have been described by Borsello and colleagues in 2003 (Borsello et al., 2003a). The neuroprotective efficacy of D-JNKI1 has been already extensively proved, both in *in vitro* and *in vivo* studies: its anti-apoptotic role was demonstrated in the treatment of different brain pathological conditions and diseases, such as cerebral ischemia (Borsello et al., 2003a; Repici et al., 2007), neuropathic pain (Manassero et al., 2012), epilepsy (Spigolon et al., 2010) and Alzheimer disease (Sclip et al., 2011, 2013, 2014).

By injecting D-JNKI1 in SMNΔ7 pups, we expected to counteract MN cell death, with the aim to reduce the progressive neurodegeneration and atrophy occurring in SMA.

## Materials and Methods

### Animals

SMN2+/+; SMNΔ7+/+; SMN+/− mice (stock number 005025; Jackson Lab, Bar Harbor, ME, USA) were bred to obtain the experimental animals, i.e., SMN−/− (SMA, as model of type II SMA) and SMN+/+ (WT) offspring. Pups were tail snipped at postnatal day 0 (P0) for identification, and genotyped by PCR assay (Valsecchi et al., 2015). SMA and WT pups were left in the cage with the mother until the sacrifice at P12. Another group of SMA mice were used for survival analysis. Animals had free access to food and water, and were kept into regular cages under 12/12-h light/dark cycle. All efforts were made to minimize the number of animals used and the suffering levels. Pups of both sexes were used in this study. The experimental procedures involving live animals were performed in strict accordance to the European Communities Council Directive 86/609/EEC (November 24, 1986) Italian Ministry of Health and University of Turin institutional guidelines on animal welfare (law 116/92 on Care and Protection of living animals undergoing experimental or other scientific procedures; permit number 17/2010-B, June 30, 2010). Additionally, the *ad hoc* Ethical Committee of the University of Turin specifically approved this study. A total of 56 SMA and 29 WT mice were used.

### D-JNKI1 Molecule and Peptide Administration

The JNK-inhibitor is a cell-penetrating peptide that selectively blocks the access of JNK to c-Jun and the other JBD-domain substrates by a competitive mechanism, as described in Borsello et al. (2003a). More in details, this inhibitor peptide was obtained by linking the 10-amino acid HIV-TAT sequence that directs cellular import to the 20-amino-acid JNK-binding motif (JBD_20_) of JNK-interacting protein-1/islet-brain 1 (JIP-1/IB1), which shows a similar binding motif of c-Jun, but has a 100-fold higher affinity (Bonny et al., 2001; Borsello et al., 2003a). WT and SMA animals were divided into PBS- and D-JNKI1-treated groups. Treated animals intraperitoneally received D-JNKI1 peptide diluted in PBS (0.3 mg/kg; D-JNKI1 group; range of injected volume: 5–30 μl, depending on age and weight; Spigolon et al., 2010; Manassero et al., 2012), while control mice received PBS. D-JNKI1 peptide/PBS were injected every 3 days, starting from P1.

### Behavioral Assessment

Behavioral tests, specifically designed for neonatal rodents (El-Khodor et al., 2008), were performed at different time points: P2, P4, P7, P10, P12 on WT and SMA mice, of both control (PBS treated) and D-JNKI1 treated groups (*N* = 3 WT PBS; 22 SMA PBS; 11 WT D-JNKI1; 34 SMA D-JNKI1). Animals were observed one at a time and then placed on a heated pad (37°C) until all the SMA and WT pups of the litter had been tested. All pups were then mixed with the cage bedding in order to minimize maternal rejection after handling and then returned to their mother. Body weight was measured before the tests, using a standard small animal balance. Four behavioral tests were performed on pups following protocols:

- *Tail Suspension Test*: in the tail suspension test pups were suspended by the tail for 15 s and a score was assigned to their hind-limb posture, from 4 (normal hind-limb spread open) to 0 (hind-limb always closed together with clasping).
- *Righting Reflex*: pups were placed on their backs on a flat surface and their failure or success in repositioning themselves on dorsal side up was assessed over 30 s.
- *Hind-limb suspension test*: this test allowed to evaluate the strength/weakness of the hind-limbs and the fatigue in pups. They were placed head down, hanging by the hind-limbs in a plastic 50 ml centrifuge tube with a cotton ball cushion at the bottom for protection. The latency to fall from the edge of the tube was evaluated and a score was assigned to the hind-limb posture, as in the tail suspension test described above. The test was performed in two consecutive trials and the average latency and score were calculated.
- *Negative Geotaxis*: for the evaluation of motor coordination and vestibular sensitivity, pups from P4 were placed on an inclined surface (approximately 35° inclination) with the head facing down. The ability of the pups to turn around and climb upwards was evaluated within 60 s and recorded.

### Tissue Preparation

For the histological analysis of the spinal cord, at P12 animals (*N* = 3 WT PBS; 17 SMA PBS; 6 WT D-JNKI1; 29 SMA D-JNKI1) were anesthetized by gaseous anesthesia and perfused transcardially with phosphate buffer (0.1 M PB, pH 7.4), followed by cold 4% paraformaldehyde (PFA) in 0.1 M PB (pH 7.4). The spinal cord was removed from the vertebral column at the lumbar level (L1–L4) and postfixed in 4% PFA for 2 h. The tissue was then cryoprotected in 30% sucrose solution in 0.1 M PB buffer overnight, then embedded, and frozen in cryostat medium (Killik, Bio-Optica, Milan, Italy). The spinal cord was cut into transverse, 40 μm thick, free-floating sections that were stored in an antifreeze solution (30% ethylene glycol, 30% glycerol, 10% PB; 189 mM NaH_2_PO_4_; 192.5 mM NaOH; pH 7.4) and stored at −20°C until being used.

For the histological examination of quadriceps muscles, another cohort of pups (five animals per group) were sacrificed by cervical dislocation. Fresh quadriceps muscles were rapidly collected, embedded in cryostat medium and cut on the cryostat. Coronal and parasagittal muscle slices (20 μm thick) were cut and collected directly onto 4% gelatin-coated glasses.

### Immunohistochemistry

For immunofluorescence staining, after rinsing in PBS to remove the antifreeze solution, the sections were incubated in a mixture of primary antibodies diluted in 0.1 M PBS, pH 7.4, 0.5% Triton X-100, and appropriate 2% normal sera for 24 h at 4°C. The following primary antibodies were used: anti-glial fibrillary acidic protein (anti-GFAP; rabbit, DAKO Cytomation, Agilent, Santa Clara, CA, USA), anti-phosphorylated c-Jun (rabbit phospho S73; Abcam), anti-neurofilament (anti-NF) H, clone SMI32 (mouse, BioLegend, San Diego, CA, USA), anti-NF (mouse, 2H3 clone, Hybridoma Bank, Iowa, IA, USA), anti-Cleaved Caspase-3 (Asp175; rabbit, Cell Signaling Technology, Danvers, MA, USA).

Then, the sections were incubated with appropriate fluorochrome-conjugated secondary antibodies (Alexafluor, anti-rabbit 647, anti-mouse 488; Cy^TM^3 AffiniPure anti-mouse and anti-rabbit; Jackson ImmunoResearch Laboratories, West Grove, PA, USA), mounted on 2% gelatin-coated slices, coverslipped with the anti-fade mounting medium Mowiol and analyzed with a Leica TCS SP5 confocal laser scanning microscope (Leica Microsystems).

For detecting phosphorylated-c-Jun (p-c-Jun) and cleaved Caspase-3 antibody co-localization, in order to solve the problem that the two antibodies were made in the same species (rabbit), p-c-Jun signal was first revealed by using the anti-rabbit IgG biotinylated secondary antibody (Vector Laboratories, Burlingame, CA, USA), followed by HRP-streptavidin incubation (diluted 1:100 in 0.1 M PBS), and the sequential TSA reactions with FITC-tyramide, 1:100 in its amplification diluent (TSA^®^ biotin detection kit, PerkinElmer, Waltham, MA, USA; Stack et al., 2014; Faget and Hnasko, 2015); then cleaved Caspase-3 signal was detected using the standard protocol for immunofluorescence described above.

For the analysis of neuromuscular junctions (NMJs), the muscle slices were incubated for 30 min at room temperature with the Alexafluor-555-conjugated bungarotoxin (Invitrogen), diluted in 0.1 M PBS, pH 7.4, 0.5% Triton X-100.

### Histochemistry

For Nissl staining, spinal cord sections were mounted on 2% gelatin-coated slides and air-dried overnight. Sections were then hydrated in distilled water, immersed in 0.1% Cresyl violet acetate (Sigma Aldrich) and cover-slipped with Eukitt (Bio-Optica).

For hematoxylin/eosin (H/E) staining, sections of quadriceps muscle were stained firstly with hematoxylin, then with eosin (Bio-Optica), dehydrated in ascending series of ethanol (95%–100%) and cleared in xylene. The sections were drawn and analyzed by Neurolucida software (MicroBrightField Inc., Williston, VT, USA) and data were obtained by the associated data analysis software NeuroExplorer (MicroBrightField).

### Western Blot

For western blot analysis, five animals for each group were rapidly killed at age P12 and L1–L4 spinal cord segments were quickly removed and frozen in liquid nitrogen; they were mechanically homogenized in a hypotonic buffer containing a mixture of proteinase and phosphatase inhibitors (Sigma-Aldrich, St. Louis, MO, USA). Protein concentrations were quantified using the Bradford Assay (Bio-Rad Protein Assay 500-0006, Munich, Germany). Five grams of TIF extracted proteins were separated by 10% SDS polyacrylamide gel electrophoresis. PVDF membranes were blocked in Tris-buffered saline (5% non-fat milk powder, 0.1% Tween20, 1 h, room temperature). Primary antibodies were diluted in the same buffer (incubation overnight, 4°C) using: c-Jun (cat. #9165, 1:1,000, Cell Signaling Technology, Danvers, MA, USA; molecular weights 48 kDa and 43 kDa), p-c-Jun [Ser63] (cat. #9164, 1:1,000, Cell Signaling Technology, Danvers, MA, USA; molecular weight 48 kDa), cleaved Caspase-3 (asp175; cat. #9661, 1: 250, Cell Signaling Technology; molecular weights 19 kDa and 17 kDa), Actin (1:5,000, Millipore; molecular weight 43 kDa). Blots were developed using horseradish peroxidase-conjugated secondary antibodies (Santa Cruz Biotechnology) and the Clarity Western ECL Blotting Substrates (Bio-Rad). Western blots were quantified by densitometry using ImageLab 6.0 software (software associated to ChemiDoc MP images, Bio-Rad) and was based on at least three independent experiments.

### Quantitative Analysis

The p-c-Jun positive MNs were quantified by counting the percentage of p-c-Jun+/SMI32+ double labeled cells on the total SMI32+ MN cell population, using confocal images (40× magnification, 1 μm z-step size, 10 μm z-volume, acquisition speed 100 Hz, format 1024 × 1024 pixels) of the ventral horns of the lumbar spinal cord. Four animals were analyzed for each group, four spinal cord slices evaluated for each animal.

For the analysis of astrogliosis in the spinal cord, confocal images (40× magnification, 1 μm z-step size, 8 μm z-volume, acquisition speed 100 Hz, format 1024 × 1024 pixels; eight fields for each animal of the spinal cord ventral horns) of GFAP-positive cells were converted in black and white images, then the density of immunopositive profiles was quantified for the four group of animals (WT PBS/D-JNKI1, SMA PBS/D-JNKI1; three animals per group).

For the analysis of NFs and NMJ innervation, confocal stacks (serial sections of 1 μm of thickness to scan the entire NMJ) were analyzed using IMARIS software (Bitplane): for each animal, at least 100 NMJs were imaged and reconstructed in 3D by Imaris software; thus, the number of NF-positive fibers in contact with each NMJs was evaluated and counted. NF accumulation in NMJs was evaluated using Imaris software, by calculating the ratio between NF labeling area/volume and bungarotoxin (NMJ) labeling area/volume. NMJ volume was evaluated using Imaris (Bitplane): more than 100 NMJs were considered for each animal. These images were used for analysis or assembled into extended-focus photographs. Brightness, color, and contrast were balanced and assembled into panels with Inkscape (Free vector graphics editors).

To morphologically evaluate the quadriceps muscle, in terms of mean fiber area and Feret’s diameter, the sections were drawn and analyzed by Neurolucida software (MicroBrightField Inc., Williston, VT, USA) and data were obtained by the associated data analysis software NeuroExplorer (MicroBrightField). More than 100 fibers were drawn and analyzed for each animal: we then averaged the means obtained from single animals.

For the MN counting (SMA PBS *n* = 6; SMA D-JNKI1 *n* = 6; WT PBS *n* = 5; WT D-JNKI1 *n* = 5), Nissl-stained slices, containing a representative series of the lumbar spinal cord (L1–L4; 1 sections every 320 μm), were analyzed using a stereological technique, the Optical Fractionator, a computer-assisted microscope and the StereoInvestigator software (MicroBrightField Inc., Williston, VT, USA). MNs were counted when characterized by an area ≥80 μm^2^ and located in a congruent position. The cell density was reported as MN number/mm^3^.

### Statistical Analysis

The data were evaluated as means ± standard error of mean (SEM). Statistical analysis was performed using GraphPad Prism 6.0 software (GraphPad Software, San Diego, CA,USA).

For the histological and histochemical analyses, we first tested the value distribution with Kolmogorov-Smirnov and Shapiro-Wilk tests of normality: then, according to their gaussian or not-normal distribution, unpaired Student’s *t*-test or Wilcoxon-Mann-Whitney test were respectively used, for simple 1:1 comparison (WT PBS vs. SMA PBS, and SMA PBS vs. SMA D-JNKI1 groups). Additionally, one-way ANOVA was used for multiple comparisons in case of parametric data.

In the behavioral analysis, the one-way ANOVA for repeated measures and the Bonferroni *post hoc* tests were used for the tail suspension test, the hind-limb suspension test, the righting reflex and the negative geotaxis test. Moreover, to better confirm the results, in those tests expressed with not normally distributed data (i.e., righting reflex and negative geotaxis test) the contingency table analysis (Fisher exact test) was applied.

The lifespan/survival of SMA PBS and SMA D-JNKI1 animals was analyzed using Kaplan-Meier test. For the body weight analysis, we used two-way ANOVA repeated measures; moreover, as suggested by El-Khodor et al. (2008), data were also represented as a Kaplan-Meier plot, defined as the time from one initiating event (birth, P0) to a terminating event: the latter one is considered as the “the postnatal day when: (1) the animal lagged in body weight by two standard deviations from the established normal average body weight gain of the WT controls; and (2) the body weight remained two standard deviations below the average on each subsequent days” until the last observation (P12; El-Khodor et al., 2008). Differences were considered significant when *p* < 0.05. The analyses were performed blinded for the genotype and the treatment of the mice.

## Results

### Analysis of Motor Neuronal and Muscular Alteration in P12 SMA Mice Compared to WT Littermates

**Figure 1:**
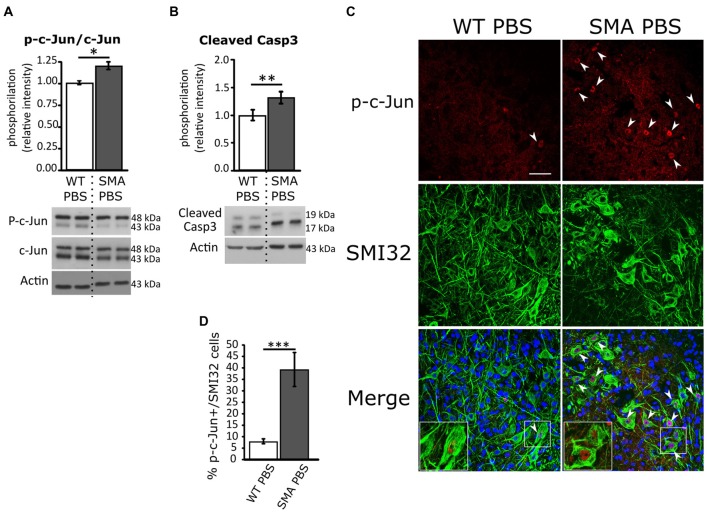
Analysis of c-Jun and active Caspase-3 phosphorylation in WT and spinal muscular atrophy (SMA) pups. **(A)** Western Blot of the relative phosphorylation intensity of phosphorylated-c-Jun (p-c-Jun)/c-Jun signal in P12 WT and SMA PBS-treated animal spinal cord. **(B)** Western Blot of the relative phosphorylation intensity of active Caspase-3 signal in P12 WT and SMA PBS-treated animal spinal cord. Unprocessed original scans of blots are shown in Supplementary Figure S1. **(C)** Confocal images showing p-c-Jun/SMI32-positive cells in the ventral horns of PBS-treated WT and SMA spinal cord. Upper panels show p-c-Jun positive nuclei (in red), indicated by arrowheads; central panels indicate SMI32-positive motor neurons (MNs; in green). In lower merge panels, the colocalization of p-c-Jun and SMI32 is displayed and, in addition, cell nuclei are labeled by DAPI staining (in blue): insets show higher magnification of p-c-Jun/SMI32-double labeled MNs. Scale bar 50 μm. **(D)** Quantification of the percentage of MNs (SMI32+) expressing p-c-Jun in WT and SMA PBS-treated animals at age P12. *P < 0.05; **P < 0.01; ***P < 0.001.

To shed light on the cell death type ongoing in SMA and understand the potential role of JNK pathway in the disease pathogenesis, by WB analysis we first quantified the p-c-Jun protein fraction (a JNK target) in spinal cords of P12 SMA and WT pups: we found an increase of about 20% in relative phosphorylation intensity in SMA group compared to WT (*p* = 0.014, Figure 1A), confirming the results of Genabai et al.’s (2015) group. Moreover, the quantification of cleaved Caspease3 relative phosphorylation showed an increase of about 32% in SMA mice (*p* = 0.003; Figure 1B). Then we also performed double immunolabeling for p-c-Jun and SMI32 protein (a specific marker of MNs) to quantify the percentage of p-c-Jun expressing SMI32-positive MNs in the ventral horns of the spinal cord. WT animals showed a small percentage (about 8%) of p-c-Jun activated MNs, indicating a physiological activation of stress signaling pathways in healthy tissue, probably due to postnatal axonal refinement (Qu et al., 2013; Yuan et al., 2014). On the contrary, SMA control mice (PBS-treated) showed a significant increase in p-c-Jun+/SMI32+ expressing cells (39%) compared to WT (Mann-Whitney test; *p* = 0.0002; Figures 1C,D). Moreover, we found that in many MNs, the expression of p-c-jun colocalized with the expression of cleaved Caspase-3, reinforcing the hypothesis that those MNs with high activation of stress signaling pathway are in the process of apoptosis (Supplementary Figure S2).

**Figure 2:**
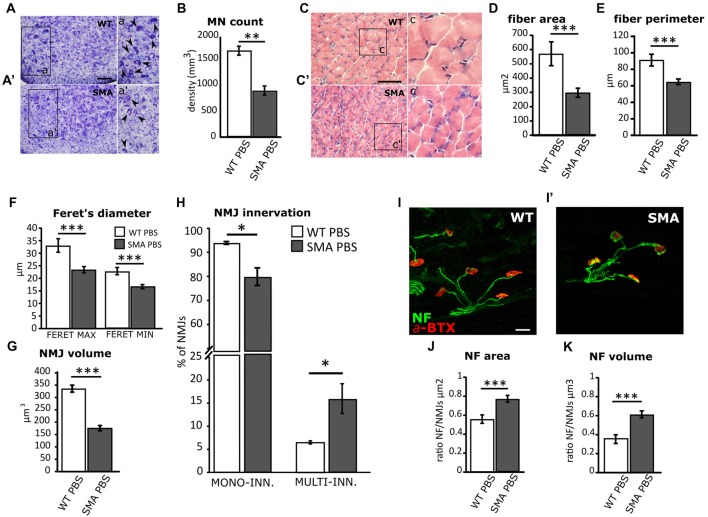
Comparison of MN survival and muscular fiber dimension and innervation between WT and SMA mice at P12. **(A,A’)** Nissl-stained MNs in the ventral horns of WT and SMA mice spinal cord. Arrowheads indicate MN in the higher magnification insets a,a’. **(B)** Quantification of MN density between WT and SMA mice. **(C,C’)** Hematoxylin/eosin (H/E)-stained representative images showing WT and SMA quadriceps fibers. Insets c,c’ show higher magnification of the muscular fibers. **(D)** Analysis of muscle fiber area in WT and SMA mice. **(E)** Analysis of muscle fiber perimeter in WT and SMA mice. **(F)** Analysis of maximal and minimal Feret’s diameter of WT and SMA muscle fibers. **(G)** Quantification of neuromuscular junction (NMJ) volume. **(H)** Quantification of the percentage of mono and multi-innervated NMJs in WT mice and SMA littermates. **(I,I’)** Double immunostaining against α-bungarotoxin (BTX, in red) and neurofilament (NF, in green) is employed for analyzing NF accumulation into the plaques in WT and SMA mice. **(J)** The area of NF accumulation is measured as the ratio between the area of the NF labeling and the area of the NMJ labeling. **(K)** The volume of NF accumulation is measured as the ratio between the volume of NF labeling and the volume of NMJ labeling. Scale bars: 50 μm **(A,A’)**; 40 μm **(C,C’)**; 25 μm **(I-I’)**. *P < 0.05; **P < 0.01; ***P < 0.001.

According to the literature (Branchu et al., 2013; Biondi et al., 2015; Genabai et al., 2015), these data confirm that JNK-signaling pathway is activated in SMA MNs and is probably involved in neuronal cell death. Indeed, confirming previous observations (d’Errico et al., 2013; Piras et al., 2017), here we report the reduction in the density of MNs in SMA spinal cord compared to WT group (WT PBS = 1,651.14 ± 90.3 cells/mm^3^; SMA PBS = 891.22 ± 82.5 cells/mm^3^; Mann-Whitney test, *p* = 0.0043; Figures 2A,A′,B). As a consequence of neurodegeneration, proximal muscles of SMA mice are early affected by atrophy (Valsecchi et al., 2015; Boido et al., 2018): indeed, quadriceps fibers showed reduced area (WT PBS = 578.4 ± 80.8 μm^2^; SMA PBS = 297.7 ± 31.7 μm^2^; unpaired *t*-test, *p* = 0.043; Figures 2C,C′,D), perimeter (WT PBS = 90.8 ± 7.1 μm; SMA PBS = 76.2 ± 1.9 μm; unpaired *t*-test, *p* = 0.036; Figure 2E), maximal (WT PBS = 32.4 ± 1.7 μm; SMA PBS = 28.04 ± 0.78 μm; unpaired *t*-test, *p* < 0.001) and minimal Feret’s diameter (WT PBS = 22.8 ± 1.4 μm; SMA PBS = 16.7 ± 0.85 μm; unpaired *t*-test, *p* < 0.001; Figure 2F). As expected, also the NMJs are strongly affected in SMA, showing a reduced endplate volume (WT PBS = 335.8 ± 14.2 μm^3^; SMA PBS = 175.7 ± 10.6 μm^3^; unpaired *t*-test, *p* < 0.0001; Figure 2G), together with an increased number of multi-innervated NMJs (WT PBS = 16.5 ± 2.3%; SMA PBS = 6 ± 0.45%; Mann-Whitney test, *p* = 0.012) and a decreased number of mono-innervated NMJs (WT PBS = 91.2 ± 0.4%; SMA PBS = 77.7 ± 2.5%; Mann-Whitney test, *p* = 0.013; Figure 2H). By Imaris software, we also quantified NF accumulation (a hallmark of the disease), by measuring the ratio between the area/volume of NF expression and the area/volume of the α-bungarotoxin (BTX)-labeled NMJs: we detected a significant increase of NF area (WT PBS = 0.55 ± 0.04 μm^2^; SMA PBS = 0.77 ± 0.03 μm^2^; unpaired *t*-test, *p* = 0.0004; Figures 2I,I′,J) and volume (WT PBS = 0.35 ± 0.04 μm^2^; SMA PBS = 0.61 ± 0.04 μm^3^; unpaired *t*-test, *p* = 0.0001; Figure 2K) in SMA NMJs compared to WT littermates.

Overall, these data suggest that JNK-signaling pathway is activated in SMA, can trigger MN death and consequently muscular/NMJ defects: therefore, the pharmacological inhibition of this pathway could lead to the amelioration of the pathology.

### D-JNKI1 Administration in WT Mice Does Not Affect the Correct Murine Development

Given the importance of JNK as a key molecule in CNS development (Coffey, 2014), we have first verified that D-JNKI1 administration did not interfere with the physiological MN development of pups: therefore we treated WT mice, by administering D-JNKI1 or PBS every 3 days, from P1 to P10. In the histological analysis of the spinal cord at P12 (day of sacrifice), we found no differences between PBS- and D-JNKI1-treated WT mice, in terms of MN density and astrogliosis (*p* > 0.05). Moreover, in the analysis of quadriceps, we found neither differences in fiber dimensions (area, perimeter and Feret’s diameters), nor in NMJ volume (*p* > 0.05). Finally, as observed for control PBS-treated, WT pups treated with D-JNKI1 peptide showed a higher percentage of mono-innervated NMJs, compared to multi- and denervated ones, but no difference in NMJ innervation percentage (*p* > 0.05). All the results are summarized in Table 1.

**Table 1:** Parameters analyzed in the comparison between PBS-treated and D-JNKI1-treated WT mice at P12.

	WT PBS	WT D-JNKI1
MN density (mm^2^)	1651.1 ± 90.3	1589.6 ± 54.4
Astrogliosis (% signal)	1.1 ± 0.2	0.94 ± 0.2
Fiber area (μm^2^)	570.4 ± 83.7	528.9 ± 52.8
Fiber perimeter (μm)	90.8 ± 7.1	88.7 ± 5.1
Max Feret’s diameter (μm)	33.1 ± 2.7	32.4 ± 1.7
Min Feret’s diameter (μm)	22.9 ± 1.4	22.4 ± 1.4
NMJ volume (μm^3^)	335.8 ± 14.2	342.7 ± 24.1
% Mono-innervated fibers	91.2 ± 0.4	91.0 ± 0.7

*The density of motor neurons (MNs) and the astrogliosis were evaluated in the ventral horns of WT spinal cord. In the analysis of quadriceps fibers, the fiber area, fiber perimeter and the Feret’s diameters (max and min) were analyzed. In the analysis of neuromuscular junctions (NMJs), the volume and the percentage of mono-innervated junctions were compared in PBS and D-JNKI1 WT groups. No statistical significant differences are observed between WT PBS and WT D-JNKI1 groups for all the parameters analyzed (p > 0.05)*.

**Figure 3:**
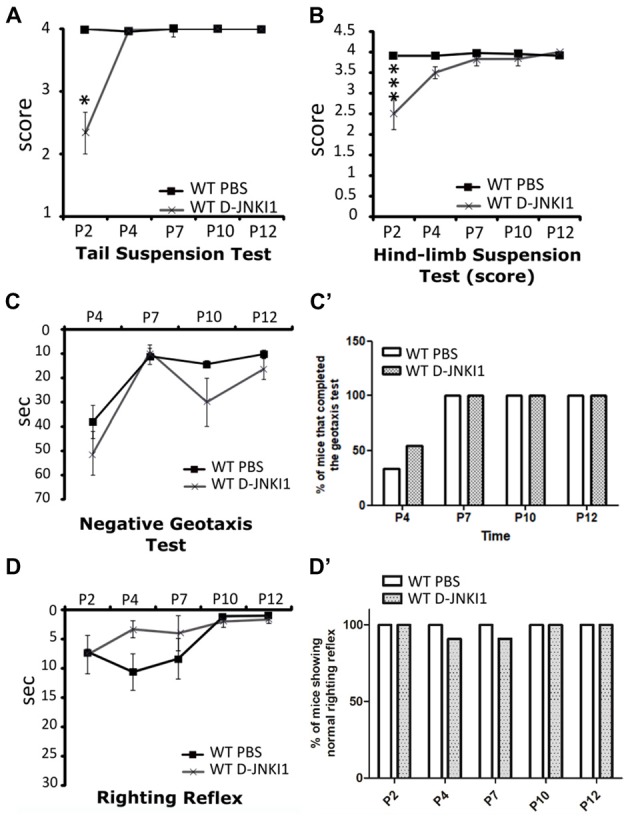
D-JNKI1 treatment partially influences motor performances only in the early phases of development of WT pups. Tail suspension test **(A)**, hind-limb suspension test **(B)**, negative geotaxis test **(C,C’)** and righting reflex test **(D,D’)** have been used to evaluate the motor performances of PBS- and D-JNKI1-treated WT mice. *P < 0.05; ***P < 0.001.

Concerning the behavioral tests, the administration of D-JNKI1 compound to WT pups seemed to slightly affect the posture in the first days after birth, as assessed by the tail suspension test (WT PBS, *N* = 3; WT D-JNKI1, *N* = 11; ANOVA for repeated measures, *F*_(1,12)_ = 16.40; Bonferroni *post hoc* test, *p* < 0.05 at P2; Figure 3A) and the hind-limb suspension test (WT PBS, *N* = 3; WT D-JNKI1, *N* = 11; ANOVA for repeated measures, *F*_(1,12)_ = 11.04; Bonferroni *post hoc* test, *p* < 0.001 at P2; Figure 3B). However, such differences were then rapidly compensated starting from P4. No differences between PBS- and D-JNKI1-treated WT were observed in the other behavioral tests (i.e., in the negative geotaxis test and righting reflex, Figures 3C–D’).

These data suggest that in WT animals the inhibition of JNK pathway could partially influence the very early postnatal development, without serious consequence for the following phases, the achievement of motor milestones and the correct MN/muscle/NMJ development.

### Reduction of Phospho-c-Jun Expression in SMA MNs After D-JNKI1 Administration

Based on the described evidence, we decided to administer D-JNKI1 inhibitor to SMA mice. The treatments were performed following the same protocol described above for WT pups. At P12, spinal cord tissue was collected and processed for WB and immunohistochemical analysis, to evaluate the effect of D-JNKI1 chronic treatment on the p-c-Jun inhibition.

**Figure 4:**
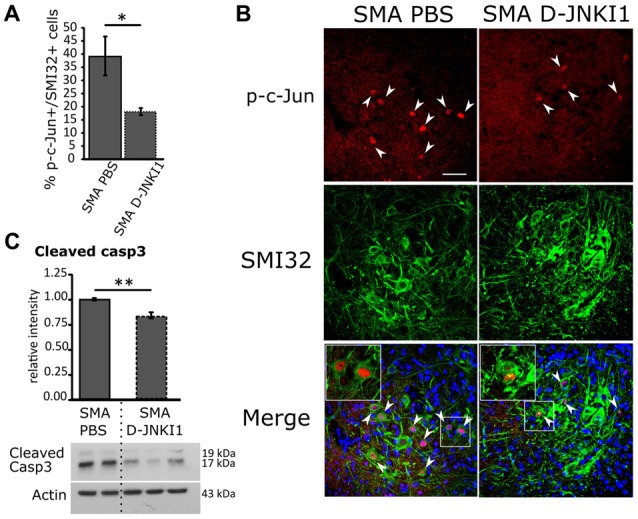
Positive effect of D-JNKI1 treatment on MN survival in SMA mice, at P12. **(A)** Quantification of the percentage of MNs (SMI32+) expressing p-c-Jun between PBS- and D-JNKI1-treated SMA pups. **(B)** Confocal images showing p-c-Jun/SMI32-positive cells in the ventral horns of PBS- and D-JNKI1 treated SMA mice. Upper panels show p-c-Jun positive nuclei (in red), indicated by arrowheads; central panels indicate SMI32-positive MNs (in green). In lower merge panels, the colocalization of p-c-Jun and SMI32 is displayed and, in addition, cell nuclei are labeled by DAPI staining (in blue): insets show higher magnification of p-c-Jun/SMI32-double labeled MNs. **(C)** Western blot of the relative phosphorylation intensity of cleaved Caspase-3 in SMA-PBS and SMA D-JNKI1 pups. Unprocessed original scans of blots are shown in Supplementary Figure S1. Scale bar 50 μm. *P < 0.05; **P < 0.01.

By WB analysis, c-Jun phosphorylation intensity was only lightly reduced in SMA animals after D-JNKI1 treatment, compared to SMA PBS (unpaired *t*-test, *p* > 0.05, data not shown). However, to better highlight the efficacy of D-JNKI1 treatment specifically on MNs, we quantified by immunohistochemical analysis the percentage of p-c-Jun expressing SMI32-positive MNs in PBS and D-JNKI1-treated SMA groups: the percentage of p-c-Jun+/SMI32+ co-labeled cells was significantly reduced in SMA mice treated with D-JNKI1 peptide (Mann-Whitney test, *p* = 0.038; Figures 4A,B).

Finally, we also evaluated by WB the expression of cleaved Caspase-3 in SMA PBS and SMA D-JNKI1: we observed a significant decrease (about 16%) in apoptotic signal after D-JNKI1 administration (unpaired *t*-test, *P* = 0.0055; Figure 4C), suggesting that JNK-inhibition can exert a neuroprotective role in the spinal cord.

### D-JNKI1-Administration Extends the Survival of MNs and Reduces Astrogliosis in SMA Spinal Cord

**Figure 5:**
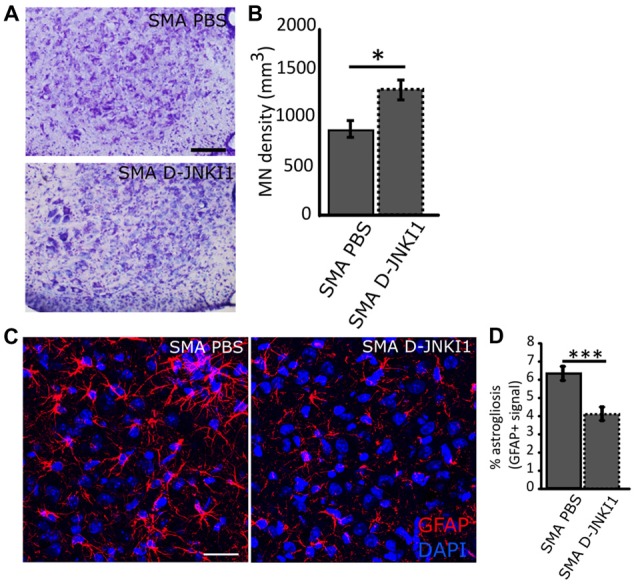
Analysis of MN survival and astrogliosis in the ventral horns of SMA spinal cord at P12, after D-JNKI1 treatment. **(A)** Nissl-stained MNs in the ventral horns showing differences between PBS-treated and D-JNKI1-treated SMA mice. **(B)** Quantification of MN density between PBS-treated and D-JNKI1-treated SMA pups. **(C)** Representative images showing a reduction in glial fibrillary acidic protein (GFAP) signal (astrogliosis) between PBS and D-JNKI1 SMA groups. **(D)** Quantification of the percentage of astrogliosis, in terms of GFAP+ signal, in the ventral horns of SMA-PBS and SMA-D-JNKI1 animals. Scale bars: 50 μm **(A)**; 40 μm **(C)**. *P < 0.05; ***P < 0.001.

To confirm the neuroprotective effect of D-JNKI1, we stereologically counted lumbar (L1–L4) MNs in treated and untreated mice. Indeed, one of the major features of SMA disease is a selective degeneration of lower MNs in the spinal cord that results in a progressive skeletal muscle denervation, atrophy and paralysis (Lorson et al., 1999). As expected, D-JNKI1-administration in SMA mice significantly increased the density of MNs, compared to PBS-treated SMA pups (SMA PBS, *N* = 6; SMA D-JNKI1, *N* = 6; Mann-Whitney test, *p* = 0.026; Figures 5A,B). Thus, these data suggest that the inhibition of JNK pathway can delay MN death in the spinal cord of SMA mice.

Moreover, in addition to the MN loss, a remarkable neuroinflammation is reported in SMA (McGivern et al., 2013; Rindt et al., 2015; Deguise and Kothary, 2017): therefore, we evaluated astrogliosis in the spinal cord by the analysis of GFAP signal in the spinal ventral horns. Results showed that D-JNKI1 treatment in SMA reduced astrogliosis compared to SMA controls (SMA PBS, *N* = 6; SMA D-JNKI1, *N* = 6; Mann-Whitney test, *p* < 0.001; Figures 5C,D). The reduction of the inflammation observed can be related to the increased MN survival.

### D-JNKI1-Treatment Reduces Atrophy of Quadriceps Muscle in SMA Mice

We have hypothesized that the MN death delay supported by D-JNKI1 could positively affect also muscular trophism and innervation. Therefore, we analyzed the morphology of quadriceps muscles at P12, by H/E staining and morphological analysis by Neurolucida Software. We evaluated the mean fiber area, perimeter, and the Feret’s diameter (max) of 100 muscle fibers per animal: we observed statistically significant differences for all the evaluated parameters in SMA mice treated with D-JNKI1, compared with SMA PBS, as reported in Table 2. Thus, we concluded that the inhibition of JNK pathway in SMA was able to slow down the atrophy of muscular fibers.

**Table 2:** Analysis of quadriceps fiber area, perimeter and maximal Feret’s diameter in PBS-treated and D-JNKI1-treated spinal muscular atrophy (SMA) mice.

	SMA PBS	SMA D-JNKI1	*t*-test
Area (μm^2^)	297.7 ± 31.7	408.3 ± 23	*P* = 0.047*
Perimeter (μm)	64.6 ± 3.4	76.2 ± 1.9	*P* = 0.040*
Max Feret’s diameter (μm)	23.4 ± 1.2	28.4 ± 0.8	*P* = 0.035*

The measurements and statistical comparisons (t-test) are indicated (*p < 0.05).

### D-JNKI1-Treated SMA Pups Show More Mature Neuromuscular Junctions

**Figure 6:**
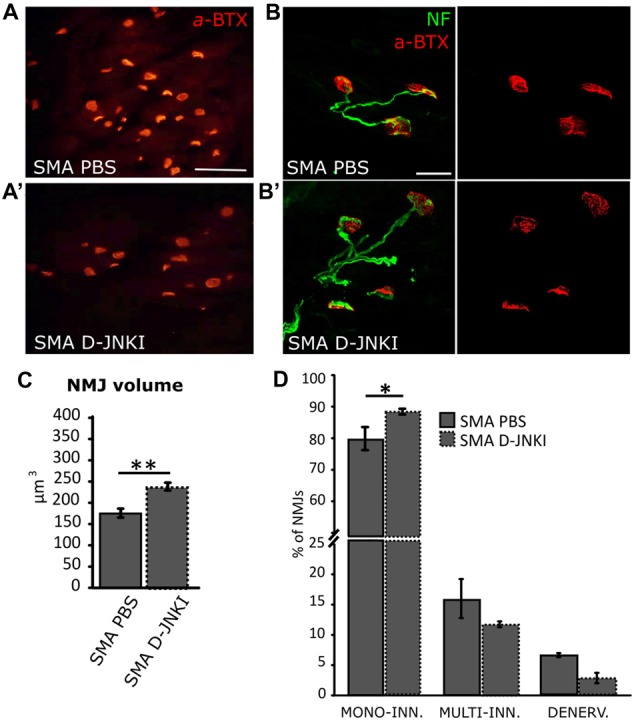
Comparison of NMJ dimensions and innervation between PBS-treated and D-JNKI1 treated SMA mice **(A–B’)** Images showing NMJ (BTX in red; Nf in green) dimensions in PBS **(A,B)** and D-JNKI1-treated **(A’,B’)** SMA quadriceps. **(C)** Analysis of NMJ volume in PBS treated and D-JNKI1 treated SMA groups. **(D)** Quantification of the percentage of mono-innervated, multi-innervated and denervated NMJs in SMA PBS and SMA D-JNKI1 groups. Scale bars: 60 μm **(A)**; 20 μm **(B)**. *P < 0.05; **P < 0.01.

Given that we observed a reduction in MN death and an increase in muscle fiber size in SMA pups after D-JNKI1 treatment, we then looked at NMJs and their innervation, to find a possible improvement in the innervation of hind-limb muscles, after inhibiting JNK pathway. We first measured NMJ volume using IMARIS software: results show that D-JNKI1 administration increased the dimensions of NMJs, compared to PBS-control SMA pups (SMA PBS, *N* = 79; SMA D-JNKI1, *N* = 121; *t*-test, *p* < 0.001; Figures 6A,A′,B,B′,C).

We also evaluated the NMJ innervation, by looking at the number of NFs contacting the endplate. Indeed, it is known that during development, the endplates are subject to a remodeling mechanism, which consists in a selective synapse stabilization due to a progressive removal of excessive motor axon terminals on myofibers, going from multiple to single innervation of each myofiber (Macintosh et al., 2006; Bloch-Gallego, 2015). Thus, the presence of mono-innervated myofibers indicates a more mature phenotype of the NMJs, together with a high perforation number and the increase of NMJ dimensions (Valsecchi et al., 2015; Boido and Vercelli, 2016). We discriminated among denervated (no filaments), mono-(contacted by only one NF) and multi-(two or more filaments) innervated NMJs in SMA. In D-JNKI1-treated SMA animals we found a significant increase in the percentage of mono innervated junctions compared to SMA controls (SMA PBS: 77.7 ± 2.5%; SMA D-JNKI1: 85.5 ± 0.87%; Mann-Whitney test; *p* = 0.028, Figure 6D), compensated by a decrease in the percentage of multi-innervated and denervated NMJs, compared to SMA PBS (Mann-Whitney test; *p* > 0.05). Thus, D-JNKI1 administration to SMA pups positively led to the acquisition of a more mature phenotype (mono-innervation) of NMJs.

Additionally, the NMJs of SMA models and patients display a massive accumulation of NF in terminal axons, due to an aberrant cytoskeletal organization of synaptic terminals, associated with defect of axonal sprouting and reduction of branched structures of the postsynaptic apparatus (Cifuentes-Diaz et al., 2002). Using IMARIS software, we found a similar level of accumulation of NF between SMA PBS and SMA D-JNKI1 groups (*t*-test for NF area and volume, *p* > 0.05, not shown), thus demonstrating that JNK inhibition has no impact on the dysregulation of the axonal transport machinery and the delayed transport of protein components to the axonal terminal.

Therefore, although we observed that the inhibition of JNK pathway in SMA mice led to a more mature phenotype of NMJs in terms of innervation (increase in mono-innervation), the treatment did not induce a reduction in the accumulation of NF.

### The Effect of D-JNKI1 Administration on Motor Behavior and Survival

To investigate whether the reduced neurodegeneration and the ameliorated muscular trophism/innervation could also improve motor performance, pups underwent a battery of behavioral tests from P2 to P12: tail suspension, righting reflex, hind-limb suspension and negative geotaxis tests. Behavioral assessment showed that D-JNKI1 administration in SMA mice improved motor performances in the tail suspension test, the hind-limb suspension test and the negative geotaxis.

**Figure 7:**
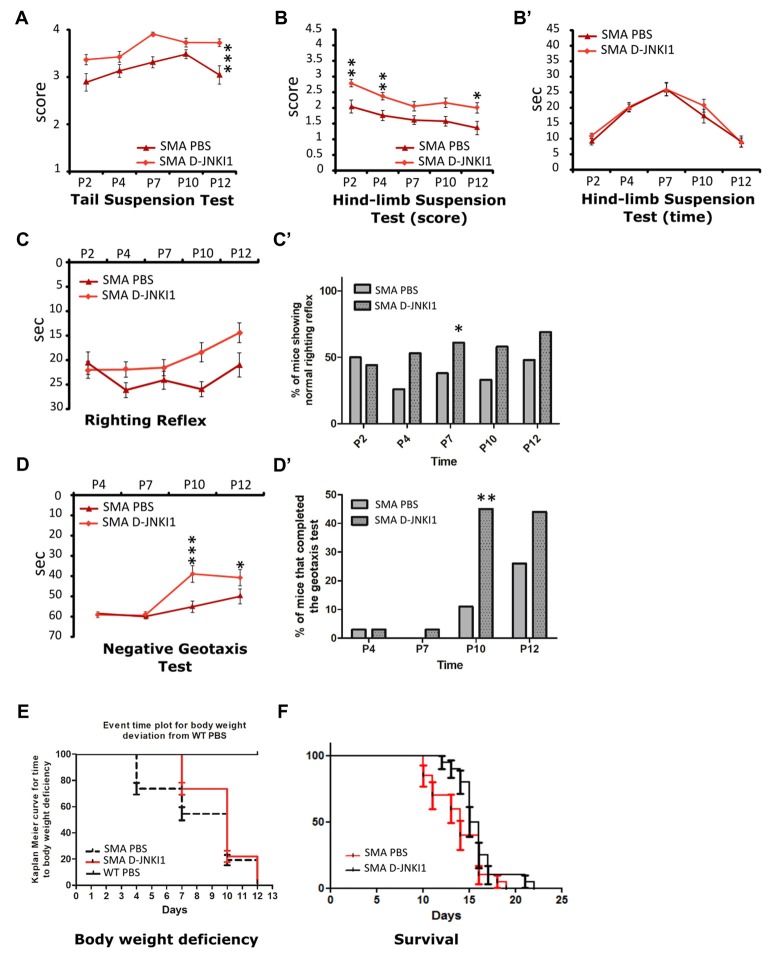
D-JNKI1 treatment improves overall state of wellbeing and motor performances in behavioral tests. Tail suspension test **(A)**, hind-limb suspension test score **(B)** and time **(B’)**, righting reflex test **(C,C’)** and negative geotaxis test **(D,D’)** have been used to evaluate the motor performances of PBS- and D-JNKI1-treated SMA mice. **(E)** Event time plot for body weight deviation of PBS SMA. D-JNKI1 SMA and PBS WT (Kaplan-Meier curve, Log-rank Mantel Cox test, *p* < 0.05). **(F)** Kaplan-Meier survival curve shows that D-JNKI1 treatment can significantly extend SMA lifespan (*p* < 0.05). *P < 0.05; **P < 0.01; ***P < 0.001.

More in detail, the administration of JNK-inhibitor ameliorated the hind-limb posture in mutant mice, leading to a higher score both in the tail suspension test (SMA PBS, *N* = 12; SMA D-JNKI1, *N* = 20; ANOVA for repeated measures; *F*_(1,30)_ = 17.22, Bonferroni *post hoc* test, *p* < 0.0001; Figure 7A) and the hind-limb suspension test (SMA PBS, *N* = 12; SMA D-JNKI1, *N* = 20; ANOVA for repeated measures; *F*_(1,30)_ = 59.71, Bonferroni *post hoc* test, *p* < 0.0001; Figure 7B). However, in the hind-limb suspension test, no differences were observed regarding the latency to fall (*p* > 0.05, Figure 7B’). No statistical significant differences in the ANOVA were also seen in the righting reflex test between SMA D-JNKI1 and SMA PBS groups, even if D-JNKI1 treated animals showed a general improvement in performing this test (Figure 7C), supported by the contingency table analysis (Fisher exact test at P7, *p* = 0.04, Figure 7C’). Conversely, in the negative geotaxis test, SMA D-JNKI1-treated animals showed a significant increase in the motor performance compared to SMA PBS, starting from P10 (SMA PBS, *N* = 12; SMA D-JNKI1, *N* = 20; ANOVA for repeated measures; *F*_(1,30)_ = 38.20, Bonferroni *post hoc* test, *p* < 0.001 at P10; *p* < 0.05 at P12; Figure 7D), also confirmed by the contingency table analysis (Fisher exact test at P10; *p* = 0.009, Figure 7D’). Thus, inhibition of JNK pathway in SMA mice partially improved motor performances, possibly due to the increased MN survival and, consequently, to the ameliorated muscular tone and strength.

We also monitored the body weight during the entire treatment. Compared to WT, the weight of SMA mice reached a plateau between P8 and P10, then started to decrease until death (Boido et al., 2018). Administration of D-JNKI1 resulted in a slight increase in weight compared to PBS-treated SMA mice (ANOVA for repeated measures. At P12, SMA PBS: 3.29 ± 0.19 g; SMA-D-JNKI1: 3.54 ± 0.14 g, unpaired *t*-test, *p* > 0.05). As suggested by El-Khodor et al. (2008), we converted the body weight analysis into a Kaplan-Meier plot: we observed that PBS-treated SMA mice showed a strongly anticipated weight reduction compared to D-JNKI1-treated group (Kaplan-Meier curve, Log-rank Mantel test, *p* < 0.0001), although the latter ones never reached the WT results (considered as control mice, Figure 7E).

Finally, in a separate set of experiments, we evaluated the D-JNKI1 efficacy in extending SMA lifespan. We observed that the administration of D-JNKI1 significantly increased the survival of treated mice, compared to PBS-treated SMA (SMA PBS, *N* = 20; SMA D-JNKI1, *N* = 20; Kaplan-Meier test, Log-rank statistical test, *p* < 0.05; Figure 7F). Additionally, the average lifespan was 13.9 ± 0.61 for PBS SMA and 15.9 ± 0.52 for D-JNKI1-treated SMA (unpaired *t*-test, *p* = 0.02).

## Discussion

### The Inhibition of JNK Cascade Represents a Potential Therapy for SMA

SMA is a severe neurodegenerative disease, the most common in infancy due to genetic causes: the autosomal recessive gene (SMN1) is carried by one person over 35/50. The major pathological landmark is a selective degeneration of lower MNs, resulting in progressive skeletal muscle denervation, atrophy and paralysis (Lorson et al., 1999; Valsecchi et al., 2015). The severity of the disease is classified into four types (SMA I-IV) based on SMN2 gene copy number, age of onset and motor function: type II SMA is considered the intermediate form, characterized by an early age of onset (7–18 months), reduced lifespan (10–40 years) and severe loss of muscular tone and strength (D’Amico et al., 2011).

Despite it is well known that the deletion/mutation in SMN1 gene is the genetic cause of SMA disease, the molecular mechanisms leading to MN death are poorly understood. Recently, it has been demonstrated that the intracellular stress signaling pathways are selectively activated in presence of low levels of SMN (Genabai et al., 2015): indeed, the upstream MAP kinases that mediate the JNK protein activation are phosphorylated in the spinal cords of SMA II mice and SMA patients. Moreover, the *in vitro* inactivation of the JNK3 is able to reduce the degeneration of SMN-deficient neurons and to provide neuroprotection (Yamasaki et al., 2012; Genabai et al., 2015). Additionally, by deleting Jnk3 in the type II SMA pups, the authors observed a significant reduction of neurodegeneration, a partial systemic rescue of SMA phenotype, improved motor functions, and increased lifespan of SMA mice (Genabai et al., 2015).

Thus, JNK signaling pathway activation seems to be involved in MN degeneration, highlighting that this cascade can be therapeutically targeted (Borsello and Forloni, 2007). For this reason, we targeted pharmacologically the progression of SMA disease by taking advantage of a selective cell-penetrating synthetic peptide inhibitor of all the three JNK isoforms. The so-called D-JNKI1 peptide has been described as a Tat-Cargo peptide that prevents the protein-protein interaction between JNK and its JBD-depended targets by competitive mechanisms, without interfering with ATP (Bonny et al., 2001; Borsello et al., 2003a; Repici et al., 2007). D-JNKI1 resulted effective in the treatment of different pathological conditions. Indeed, in experimental models of focal cerebral ischemia, the injection of D-JNKI1 peptide was able to reduce the infarct size by about the 70%–90%: the treatment was effective both when administered as late as 12 h after ischemia (Borsello et al., 2003a) and before the ischemic lesion, in pre-treatment paradigms (Repici et al., 2007). Similarly, in an experimental model of epilepsy, D-JNKI1 administration was able to reduce the number of degenerating neurons in hippocampal CA (Spigolon et al., 2010) and to prevent ATP release and poly(ADP-ribose)-polymerase (PARP) cleavage in brain mitochondria (Zhao et al., 2012). Importantly, D-JNKI1 reverted the cognitive impairments and LTP defects in AD mouse model as well (Sclip et al., 2011). Finally, following sciatic nerve transection, the pharmacological inhibition of all the three JNK isoforms prevented the onset of neuropathic pain, by reducing the reactivation of the downstream GAP43 axonal protein after injury and by inducing a significant analgesic effect (Manassero et al., 2012).

### D-JNKI1 Administration Reduces Apoptotic Marker Expression in the Spinal Cord of SMA Mice

Both the activation of JNK and the phosphorylation of c-Jun protein have been observed in spinal cord MNs from SMA mice and SMA patients, compared to healthy subjects (Genabai et al., 2015). Our results confirm such observations, since at P12 c-Jun phosphorylation is higher in type II SMA mice compared to WT littermates, suggesting the activation of the intracellular stress signaling pathways. Thus, D-JNKI1 represents a proper peptide to be tested in SMA mice. Therefore, we chronically administered JNK inhibitor, by i.p. injection, in type II SMA pups, starting from age P1 until P10, and we analyzed c-Jun phosphorylation and cleaved Caspase-3 levels both by WB and IHC. The dose and timing of D-JNKI1 administration have been previously tested, and no toxic effects have been found on cell survival, due to the peptide concentration or to chronic administration (Repici et al., 2007; Spigolon et al., 2010). Indeed it has been showed that multiple D-JNKI1 administration show cumulative neuroprotective effects on cells (Gao et al., 2009; Manassero et al., 2012); moreover, D-JNKI1 peptide can cross the BBB with high efficiency in both intact and lesioned CNS (Borsello et al., 2003b; Repici et al., 2007): since in SMA it is still debated if BBB is intact (Lorson et al., 2010; Somers et al., 2016), D-JNKI1 administration can bypass this issue anyway.

We observed that D-JNKI1 administration can reduce p-c-Jun levels in SMA spinal cord: this was particularly evident by IHC analysis, by which we have quantified the number of p-c-Jun-positive MNs. Our results confirm a strong reduction of p-c-Jun+/SMI32+ cells after treatment, and provide evidence of the inhibitory role *in vivo* of peptide in MNs. Finally, the protective effect of D-JNKI1 was also demonstrated by the reduction in cleaved Caspase-3 signal observed in SMA spinal cord after treatment.

### D-JNKI1 Administration Exerts Neuroprotective Effects and Delays Motor Performance Worsening in SMA Mice

By quantifying the density of MNs in the spinal cord of SMA mice, we found that D-JNKI1 treatment is effectively neuroprotective against MN degeneration. In addition, the peptide significantly decreased neuroinflammation (in terms of reduction in astrogliosis) in SMA treated pups compared to SMA controls. Thus, accordingly to the literature (Genabai et al., 2015), our data strongly demonstrate that, in SMN deficiency condition, JNK activation is implied in MN degeneration, and suggest JNK inhibition as a potential therapeutic approach to reduce neuronal death in spinal cord.

In case of SMA, the consequence of the degeneration of spinal cord MNs is a progressive relentless muscular atrophy that leads to premature death due to respiratory failure (Lorson et al., 2010; Govoni et al., 2018). Thus, to evaluate whether the induced neuroprotection correlated to an increase muscular trophism, we then looked at the quadriceps, a muscle early affected by the disease. By analyzing the fibers, indeed, we found differences between D-JNKI1- and PBS-treated SMA pups, with a significant increase of muscle fiber size (in terms of mean fiber area and perimeter) in D-JNKI1-treated animals. Moreover, the evaluation of Feret’s diameters (which are highly suggested parameters to determine the size of muscle fibers; Dubache-Powell, 2008; Boido et al., 2018) confirms such differences, highlighting that the increased survival of MNs after D-JNKI1 administration leads to a reduction of muscular atrophy.

Increasing evidence in the last years suggest that the NMJs of SMA animal models and SMA patients are also altered, showing a delayed development. Indeed, several alterations have been described in SMA NMJs, including immature phenotype, reduced perforation number, small dimension, and remarkable fragmentation (Cifuentes-Diaz et al., 2002; Kariya et al., 2008; Kong et al., 2009; Valsecchi et al., 2015; Boido and Vercelli, 2016; Boido et al., 2018). Thus, we analyzed if the neuroprotective effect of D-JNKI1 could also affect NMJ development. We found that JNK inhibition results in a partial endplate rescue with improved NMJ size and innervation. Indeed, the increase in mono-innervated NMJs after D-JNKI1 treatment (with the resulting decrease in multi-innervated and denervated NMJs) suggests a more mature NMJ phenotype, that, in turn, might influence the muscle trophism (Ling et al., 2012). Therefore, the inhibition of JNK signaling pathway represents a valid target to partially rescue SMA phenotype by supporting the crosstalk between MNs and muscles, as already demonstrated in the inhibition of ROCK pathway (Bowerman et al., 2010, 2012; Genabai et al., 2015).

Moreover, SMA NMJs show a pathological accumulation of NF that is associated to a slower and delayed transport of protein components, important for the endplate maintenance and functionality (Murray et al., 2008; Torres-Benito et al., 2012; Boido and Vercelli, 2016). Although we found an improvement in the NMJ innervation, this does not correlate with a reduction in NF accumulation in treated SMA mice. Indeed, we observed only a slight reduction in D-JNKI1-treated SMA NF volume compared to PBS-treated littermates. The causes of NF accumulation are still poorly understood (Dale and Garcia, 2012), although shared by several neurodegenerative diseases, such as Alzheimer’s disease (Yang et al., 2009) amyotrophic lateral sclerosis (Brady, 1993) and symmetrical sensory polyneuropathy (Fernyhough et al., 1999). Interestingly, it seems that the increase in phosphorylation of NF subunits is concomitant with the increase in phosphorylation of JNK1/2 (Fernyhough et al., 1999; Yang et al., 2009), suggesting the hyperphosphorylation and accumulation of NF as a JNK1/2 dependent mechanism. Therefore, we expected a significant reduction of NF in D-JNKI1-treated SMA mice that inexplicably did not occur. As known, SMN deficiency affects the splicing machinery activity, probably determining alterations also in the microtubule-based motor proteins and other interacting proteins, and justifying the altered cellular trafficking and NF accumulation: due the complexity of the mechanisms involved and intertwined, it is possible that D-JNKI1 is not able to completely rescue the axonal trafficking defects observed in SMA (Ikenaka et al., 2012).

However, overall, we proved the D-JNKI1 efficacy not only histologically (at spinal and (neuro) muscular level), but also behaviorally: we found that the inhibition of JNK signaling pathway delayed the worsening of motor behavior, in particular in the tail suspension, the hind-limb suspension and the negative geotaxis test. Moreover, we found that D-JNKI1 administration on SMA pups delays the progressive weight loss, compared to SMA control littermates. Finally, JNK inhibition increased SMA mice lifespan of about 2 days, that can be considered a significant increase related to the short SMA mice lifespan (about 14 days). Nevertheless, seen the impressive histological/molecular results we obtained, we expected an even higher lifespan increase: however, the SMA pathogenesis is very complex and surely other pathways leading to MN death are implicated and can trigger the neurodegeneration (as for example the autophagic pathway or the crosstalk between Rho and ERK kinases, whose dysfunctions are well documented in SMA; Hensel et al., 2017, and Piras and Boido, 2018, respectively).

### Concluding Remarks

Although a definite cure for SMA is still missing, in the last years, the researchers focused their attention on both SMN-dependent and independent approaches, which have shown promising results in order to find effective therapeutic strategies (Govoni et al., 2018).

To stimulate the protein synthesis of SMN2 gene by enhancing transcription, many SMN-dependent approaches have been developed (Lorson et al., 2010; Howell et al., 2014; Maharshi and Hasan, 2017; Mendell et al., 2017; Talbot and Tizzano, 2017; Tosolini and Sleigh, 2017), also by modulating activators/inhibitors of different signaling pathways (e.g., ERK/ELK-1 and AKT/CREB, Biondi et al., 2010; Branchu et al., 2013; STAT5/prolactin, Farooq et al., 2011, NF-κB, Arumugam et al., 2018). SMN-independent approaches are also able to partially rescue SMA phenotype, targeting different pathways (as autophagy, Piras et al., 2017) or proteins, like ROCK (Bowerman et al., 2010, 2012), Plastin3 (Oprea et al., 2008) and the zinc finger protein ZPR1 (Ahmad et al., 2016). Moreover, some therapeutic strategies are aimed at ameliorating SMA phenotype by acting on endplates and improving neuromuscular transmission (Wyatt and Keirstead, 2010; Wadman et al., 2012; Nishimune et al., 2014; Boido et al., 2018; Lai et al., 2018).

Among the others, to counteract the progressive neurodegeneration, the inhibition of the apoptotic cascade seemed particularly interesting: indeed, the genetic inhibition of Jnk3 in SMA mice resulted in the partial restore of the phenotype without affecting the levels of SMN in the nervous system and muscles (Genabai et al., 2015). Here we demonstrated that the pharmacological inhibition of all the three isoforms of JNK proteins is useful for reducing neurodegeneration in SMA mice, without showing side effects (i.e., evident physical or psychical discomforts, premature death, interference with the MN development mechanisms…). Our results support the previous findings on Jnk genetic inhibition in SMA mice by Genabai et al. (2015), representing an implementation of their results, in the perspective of translational pharmacological approaches. Indeed, we found that the use of D-JNKI1 successfully results in a partial systemic rescue of the SMA phenotype, by reducing MN death, delaying motor function worsening, and improving muscle fiber thickness, NMJ size and innervation.

Overall, our results suggest that the deficit of SMN and JNK activation could be intertwined. It is known that in neurons JNK signaling is a Janus-faced pathway and can play a dual role, by mediating either physiological or pathological responses (Coffey et al., 2000). Indeed, on one side JNK can regulate neuronal activity by inducing phosphorylation of microtubule-associated proteins, leading to microtubule stability in neurite formation (Xu et al., 2011; Coffey, 2014); on the other, JNK is also involved in stress-induced apoptosis (Kuan et al., 1999; Tournier et al., 2001; Coffey et al., 2002), playing a critical role during injury responses associated with neurodegeneration (Xu et al., 2011; Genabai et al., 2015). Moreover, JNK may cooperate with other signaling pathways to generate context and site-specific responses (Lamb et al., 2003; Xu et al., 2011).

As concerns SMA, it is likely that the decrease in SMN level leads to cellular stress, resulting in JNK pathway phosphorylation and contributing to MN death, as proposed by Genabai et al. (2015): they reported the activation of the MAP3K proteins ASK1 and MEKK1 in the spinal cord of SMA patients and mice, suggesting that at least two possible signaling pathways can be involved in JNK activation. Indeed, they observed a marked increase in the activation of the MAP2K protein MKK4 (known to be activated by ASK1) compared to other MAP2Ks (i.e., MKK7), suggesting a specificity for the activation of the pathways downstream to JNK (Genabai et al., 2015). Moreover, in neurons, low levels of SMN may result in free pools of protein Gemin5—a part of SMN complex—which in turns may increase the phosphorylation levels of ASK1/MKK4/JNK scaffold complex. In addition, the protein complex composed by MEKK1/MKK7/JNK may be activated by the specific neuronal protein JIP3 (Genabai et al., 2015; Ahmad et al., 2016).

Thus, by D-JNKI1, that blocks the JIP-JNK interaction, both pathways might be affected. Further studies will elucidate whether a specific pathway is preferentially involved in SMA progression, in order to exploit it for more focused therapies.

Moreover, given that SMA can be considered as a multi-systemic disease (Shababi et al., 2014; Simone et al., 2016), we can hypothesize that the inhibition of all the JNK isoforms could exert protective effects also in other body district affected in SMA pathology, such as the heart (Bevan et al., 2010; Heier et al., 2010), the diaphragm (Supinski et al., 2009) and other skeletal muscles (Anderson et al., 2003), in which it has been shown that this family protein is expressed.

Finally, our treatment could represent an effective potential adjuvant to other therapies, like Nusinersen (ISIS 396443), an antisense oligonucleotide (ASO) already used in phase 3 clinical studies to increase the production of full-length SMN2 protein (Adams, 2017; Maharshi and Hasan, 2017), or AVXS-101, an AAV-based treatment to restore SMN protein expression (Mendell et al., 2017). Indeed, the use of ASOs or gene therapy represents a very promising strategy for SMA, as demonstrated by the improvement of motor functions and extended survival observed in mouse models and human patients (Parente and Corti, 2018). However such approaches have some important limitations: (i) they are merely SMN-dependent strategies and, despite their remarkable efficacy, they are not completely effective in arresting the disease progression, probably because other molecular pathways contributing to SMA pathogenesis are overlooked; (ii) they may require repeated lumbar punctures (Nusinersen) that, in case of scoliosis or vertebral fixation surgery, can represent a hard challenge for a prolonged application; (iii) their cost is extremely expensive (for Nusinersen, the expense can reach $750,000 in the first year and approximately $375,000/year subsequently; Goyal and Narayanaswami, 2018); and (iv) the majority of trials have strict eligibility criteria, determining the exclusion of milder patients.

For all these reasons, therapies targeting other molecular pathways are strongly required, in order to assure an effective treatment to all the SMA patients. Indeed, by delaying the MN death, D-JNKI1, eventually in combination with ASOs and gene therapy, could increase their SMN-dependent effects, since such therapies start when the symptoms are already present and therefore the cell death pathway already triggered.

## Author Contributions

RS, MB and AV conceived and designed the experiments and wrote the article. TB provided the inhibitor peptide and performed western blot experiments. RS and MB performed all the other experiments and analyzed the data. RS, MB, TB and AV reviewed the article.

## Conflict of Interest Statement

The authors declare that the research was conducted in the absence of any commercial or financial relationships that could be construed as a potential conflict of interest.
