# Supplementary material for: Pharmacological c-Jun NH2-Terminal Kinase (JNK) Pathway Inhibition Reduces Severity of Spinal Muscular Atrophy Disease in Mice
Source: Front Mol Neurosci. 2018 Sep 4;11:308. doi: 10.3389/fnmol.2018.00308 (PMC6131195; doi:10.3389/fnmol.2018.00308)
Supplement: Supplementary file 1 [file Data_Sheet_1.docx]

Supplementary Material

Pharmacological c-Jun NH_2_-terminal kinase (JNK) pathway inhibition reduces severity of Spinal Muscular Atrophy disease in mice.

Roberta Schellino^1#^*, Marina Boido^1,2#^, Tiziana Borsello^3,4^, Alessandro Vercelli^1,2^.

#These authors have equally contributed to this work

*** Correspondence:** Dr. Roberta Schellino**:** roberta.schellino@gmail.com

# Supplementary Figures


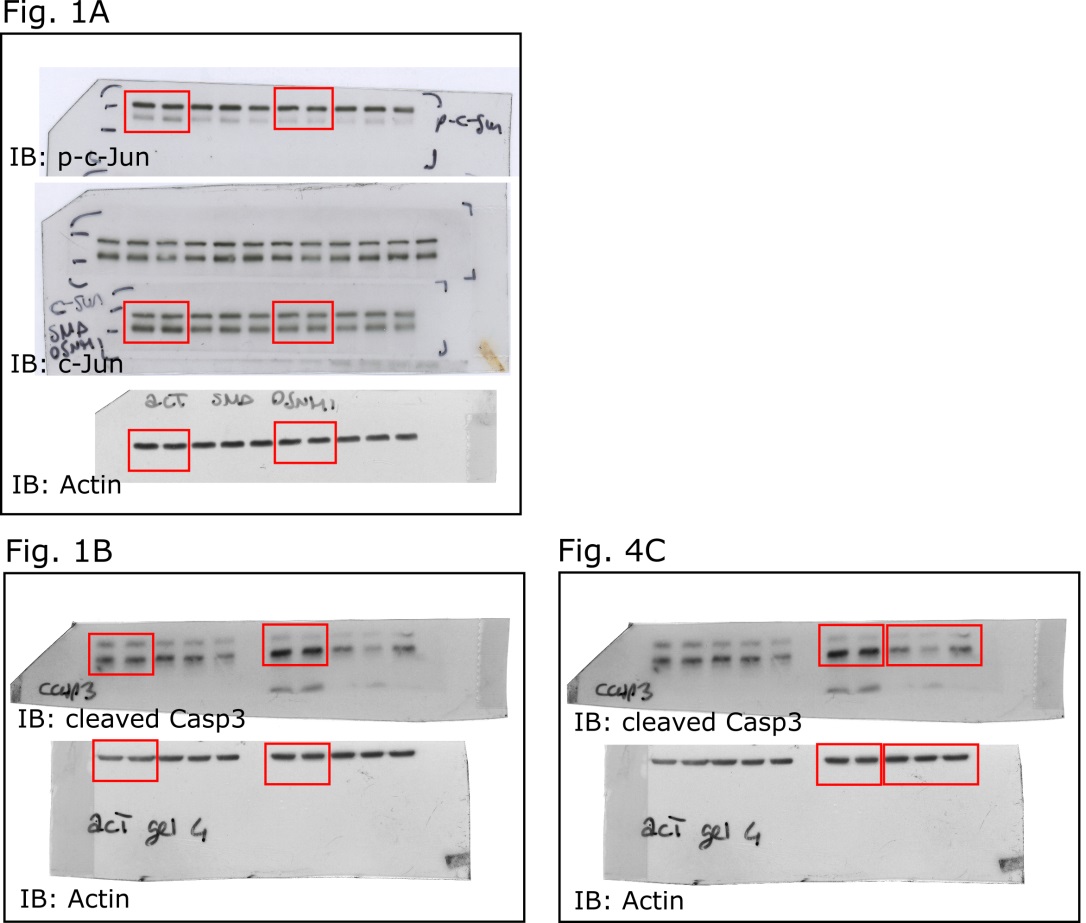


**Supplementary Figure 1.** Collection of the unprocessed blot scans used in this manuscript.


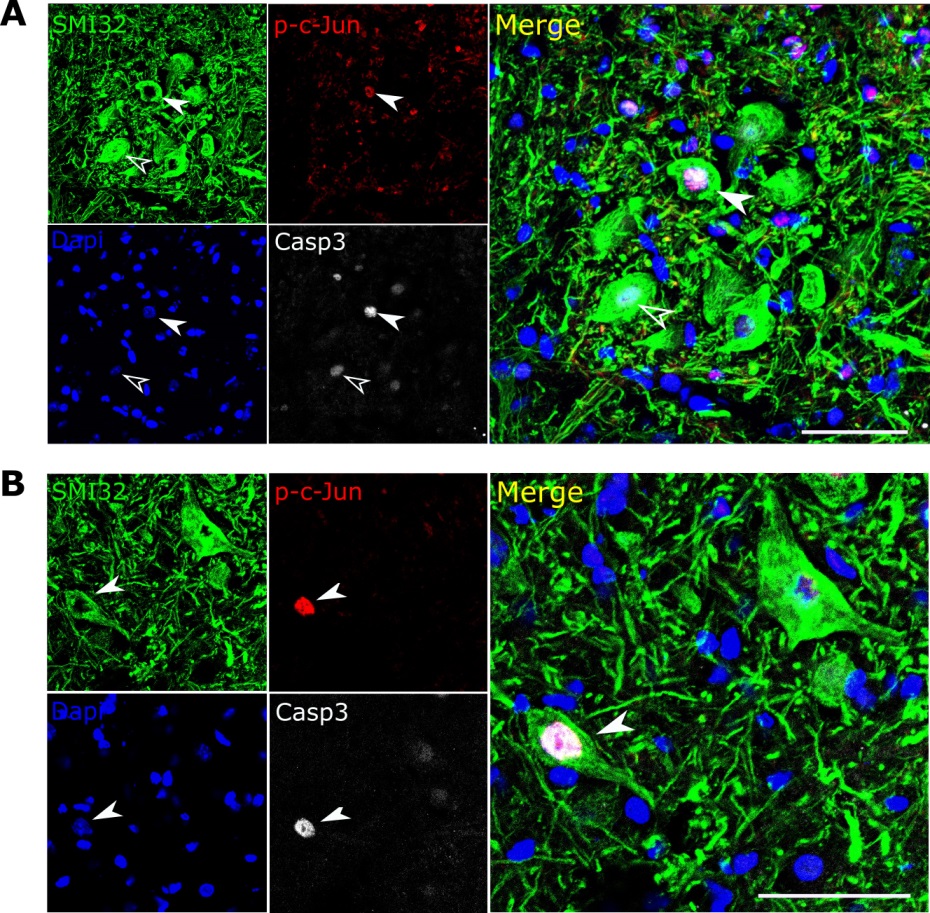


**Supplementary Figure 2.** **(A-B)**  Two examples of confocal images showing that SMA motor neurons with high activation of the stress signalling pathway are in the process of apoptosis. Full arrowheads indicate SMI32-positive motor neurons (in green) expressing both p-c-Jun (in red) and cleaved caspase3 (in white) proteins. Empty arrowhead indicates a cleaved caspase3 positive/p-c-jun negative cell. Cell nuclei are labelled by DAPI staining (in blue). Scale bar 50 µm.
